# Dynamic control of particle separation in deterministic lateral displacement separator with viscoelastic fluids

**DOI:** 10.1038/s41598-018-21827-7

**Published:** 2018-02-26

**Authors:** Yuke Li, Hongna Zhang, Yongyao Li, Xiaobin Li, Jian Wu, Shizhi Qian, Fengchen Li

**Affiliations:** 1 School of Energy Science and Engineering, Harbin Institute of Technology, Harbin, 150001 China; 2 Sino-French Institute of Nuclear Engineering and Technology, Sun Yat-sen University, Zhuhai, 519000 China; 3 Institute of Micro/Nanotechnology, Old Dominion University, Norfolk, Virginia 23529 USA

## Abstract

We proposed an innovative method to achieve dynamic control of particle separation by employing viscoelastic fluids in deterministic lateral displacement (DLD) arrays. The effects of shear-thinning and elasticity of working fluids on the critical separation size in DLD arrays are investigated. It is observed that each effect can lead to the variation of the critical separation size by approximately 40%. Since the elasticity strength of the fluid is related to the shear rate, the dynamic control can for the first time be easily realized through tuning the flow rate in microchannels.

## Introduction

A deterministic lateral displacement (DLD) array is a microfluidic particle-separation device that takes advantage of the asymmetric bifurcation of laminar flow around obstacles, which was firstly introduced by Huang *et al*.^1^ Since the invention of DLD, diverse applications have been realized in sorting and enrichment in tumor research and clinical diagnostics, e.g. purification of *Aspergillus* spores^2^, blood analysis^3–5^, detection of circulating tumor cells^6–8^, where DLD arrays are used to separate particles or cells by size from millimeter to sub-micrometer.

**Figure 1:**
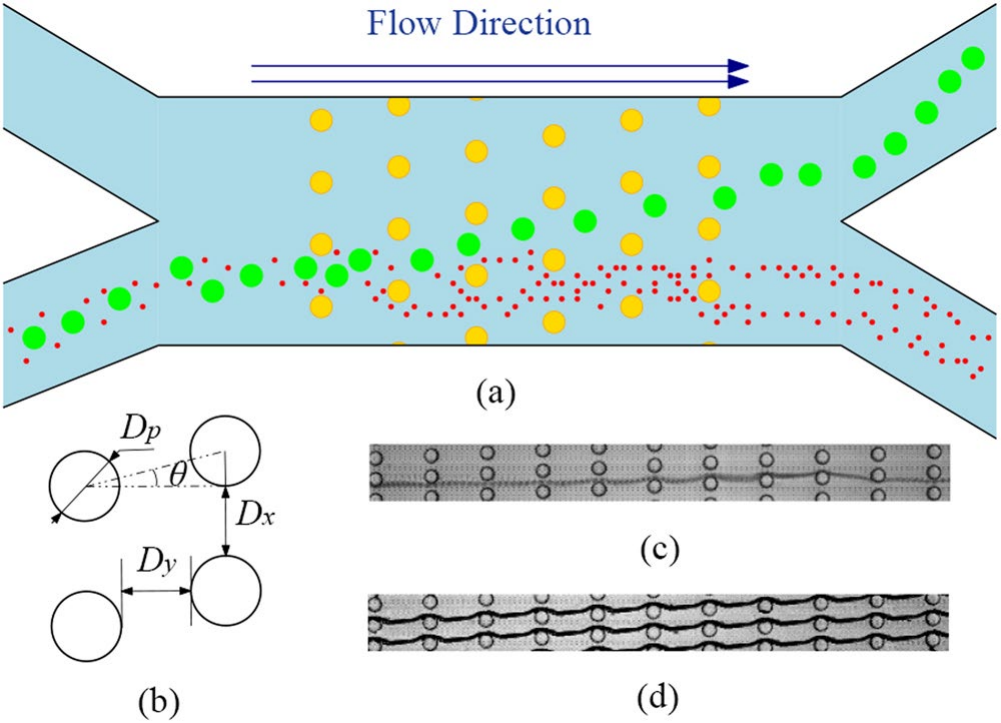
Schematics of (**a**) a DLD device with periodic arrays (yellow) and how large particles (green) and small particles (red) are separated, (**b**) a unit of rhombic posts with diameter *D*_*p*_. Superposition photos of particles behaving (**c**) *zigzag mode* (*N* = 8), (**d**) *displacement mode* (*N* = 8).

The DLD devices comprise a periodic array of micrometer-scale obstacles, which decides the separation distance of the particles with different sizes, as shown in Fig. 1(a). In a DLD device, the gap distance between two lateral posts is *D*_*x*_ and the distance along the flow direction between the nearest posts of adjacent rows is *D*_*y*_, as shown in Fig. 1(b). The basic principle can be understood by the streamline orientation of DLD arrays. Fluid emerging from the gap between two posts will encounter another post in the next row, and therefore it will bifurcate as it moves around the post. After negotiating *N* (a period) obstacles, the fluid can conceptually be divided into *N* regions. When a small particle enters the array and negotiates the posts, it will follow streams continuously, and after encountering *N* posts i.e. *N* rows, it will restore to the original direction, moving in an average flow direction matching the fluid. This particle motion is termed as “*zigzag mode*” (see Fig. 1(c) and Supplementary Video S1). However, a larger particle whose center is out of the boundary of the first stream will be displaced laterally by the obstacles into the second stream. This motion is termed as “*displacement mode*” (see Fig. 1(d) and Supplementary Video S2). By accumulating the cross-flow displacement, the larger particle will eventually migrate across the streamlines with the direction *θ*. The transition between two modes is sharp and it occurs at a critical size *D*_*c*_, about twice width of the first stream. The principle of the critical particle size implies that the particle motion in a fixed DLD array is bimodal either with diameter lower than *D*_*c*_ in “*zigzag mode*” or with diameter larger than *D*_*c*_ in “*displacement mode*”. Since each stream carries the same fluid flow, *D*_*c*_ can be analytically approximated using^9^, *D*_*c*_ = 2*αD*_*x*_/*N*, where *α* is a variable parameter to accommodate for non-uniform flow through the gap. Davis^10^ derived a power-law formula (*D*_*c*_ = 1.4*D*_*x*_*N*^−0.48^) to predict *D*_*c*_ by fitting the data collected over about 20 different devices over a wide range of *D*_*x*_ from 1.3 *μ*m to 38 *μ*m and *N* from 2 to 20.

The bimodal separation however cannot meet the need for practical applications, in which suspensions with particles of various sizes are required to operate. To this end, various advanced DLD devices were designed for multiple critical thresholds, and the corresponding methods can be regarded as passive ones and active ones. A passive DLD device with multiple critical sizes utilizes the adjustment of the configuration of posts, e.g. the shape of posts^11^, the depth of the channel^12^, the gap between the posts^13^, and hydrodynamic forces^14^, and so on. An active DLD, however, enable to tune critical diameters with external forces exerted on particles and even a live feedback setup can be realized. Several active technologies have been proposed, e.g. mechanical^15^, gravitational^16^, dielectrophoretic (DEP)^17,18^ and acoustic^19^, and so on.

In recent years, viscoelastic-based particle separation^20–24^ and focusing^25–29^ have been known as an efficient way to manipulate particles in microfluidics. By adding only small amount of synthetic polymers or biological polymers, such as DNA and hyaluronic acid, center-focusing in non-Newtonian fluids provides a new approach to manipulate different particles, including blood cells^20,21,23^, magnetic particles^27,28^, even nano-particles^22^. The normal stresses arising from fluid viscoelasticity are responsible for particle lateral migration to the narrow central core region of the channel in elasticity-dominant fluid^30–32^. The elastic lift force **F**_**e**_ scaling as ${{\bf{F}}}_{{\bf{e}}}\propto {a}^{3}\nabla {N}_{1}$^33^ (the first normal stress difference *N*_1_ = *τ*_11_ − *τ*_22_, where subscripts 1 and 2 are the direction of primary velocity and the direction of velocity variation, respectively) in inertial microfluidics suggests particle migration velocity is strongly dependent on blockage ratio and viscoelasticity of the fluid medium^34^. Inspired by **F**_**e**_ pushing particles away from sidewalls, particles may suffer extra repulsive elastic force on particles when they passing through periodic obstacles in DLD arrays. We introduced viscoelasticity of fluid medium into DLD arrays to observe peculiar phenomenon.

In this paper, we realize dynamic control of *D*_*c*_ in DLD separators by introducing viscoelastic fluids. This is for the first time to adopt viscoelastic fluids in DLD, while all previous papers are restricted to Newtonian fluids, except one to shear-thinning effects numerically^14^. One most important advantage of this technology is offering considerable control of *D*_*c*_ in a single DLD device. The peculiar rheological properties of non-Newtonian liquids, such as non-zero normal stress differences, shear-rate-dependent viscosity^35^, etc., can be exploited to design spectacular devices or improve some existing processes. Therefore, in DLD devices, the introduction of shear-rate-dependent viscosity and nonlinear elastic forces is expected to modify the critical particle size *D*_*c*_. Comparing with other active DLD devices, an obvious advantage of employing non-Newtonian fluids in DLD devices is that other auxiliary equipment is no longer required. D’Avino^14^ mainly focused on the shear-thinning fluid and observed that *D*_*c*_ declines with shear-thinning effect enhanced numerically. Here, not only shear-thinning but also elastic effects of the applied viscoelastic fluid medium on particle separation in a DLD device are performed through extensive experimental investigations. We further realize a dynamic variation of *D*_*c*_ by altering the flow rate utilizing the elasticity.

## Results

**Figure 2:**
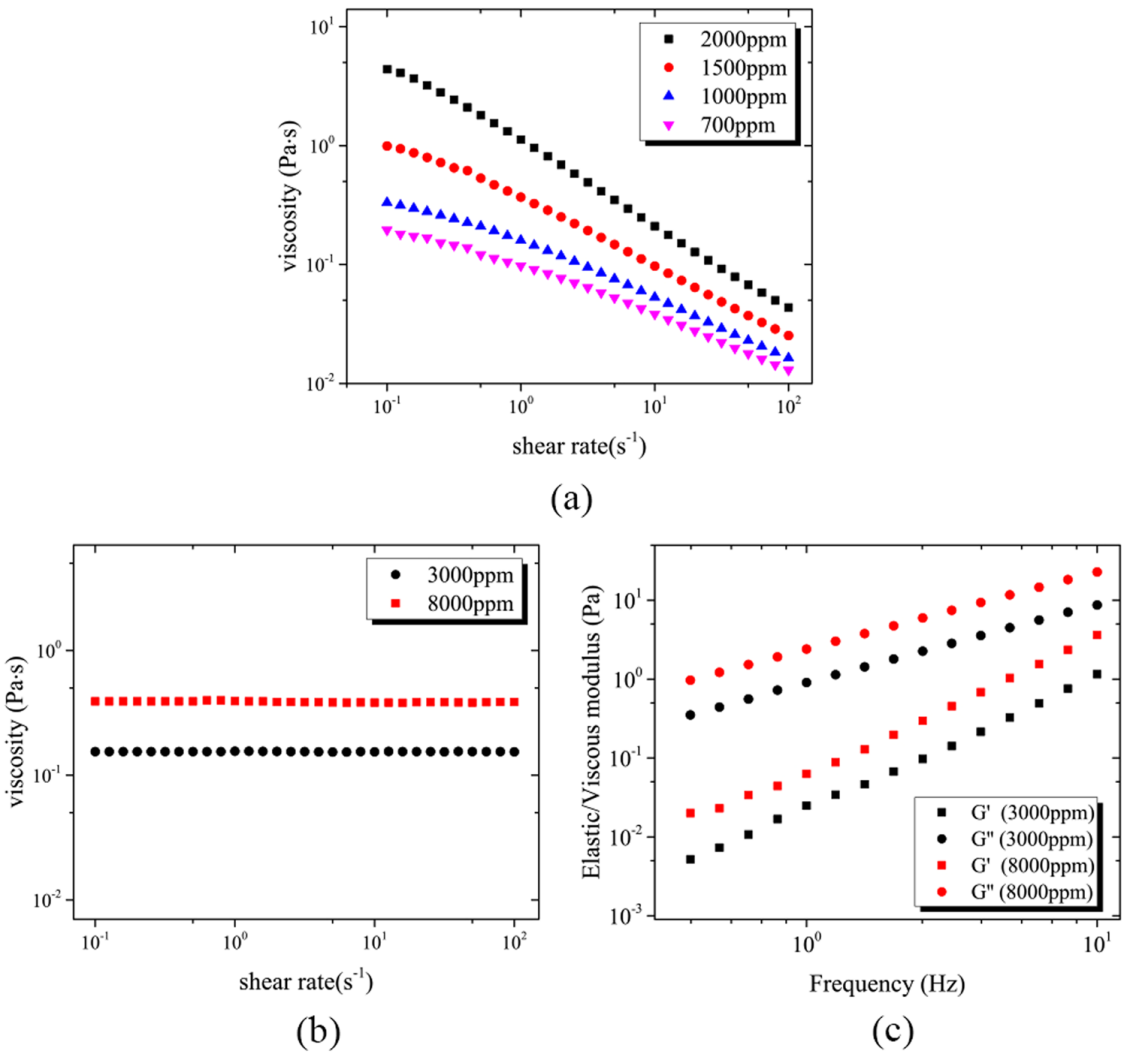
(**a**) Viscosity versus shear rate of Xanthan Gum solutions with different concentrations. (**b**) Viscosity versus shear rate and (**c**) elastic/viscous modulus versus frequency of PVP solutions with different concentrations.

**Figure 3:**
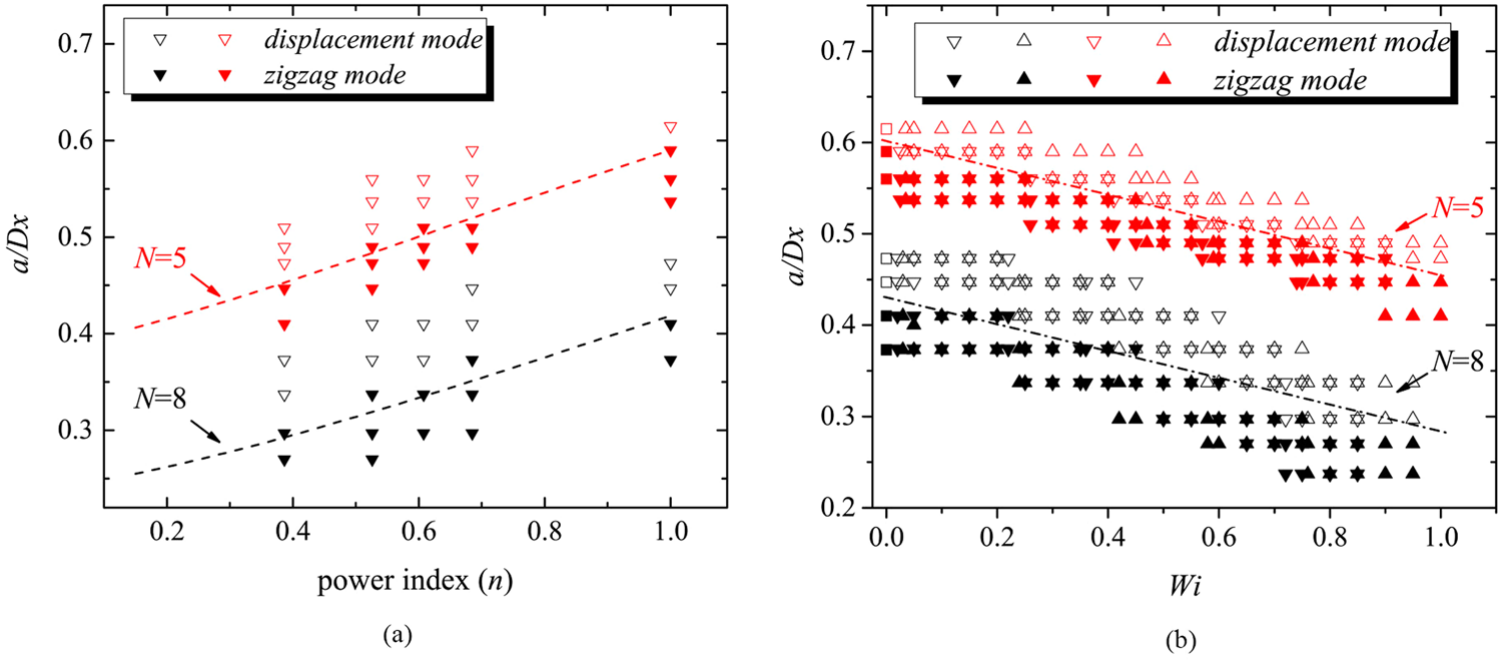
Dimensionless *a*/*D*_*x*_ versus (**a**) power index *n* and (**b**) Weissenberg number *Wi*, where *a* is the particle diameter. The diameters of particles adopted in this experiments are listed in Table 2. (**a**) The dashed lines are results predicted by numerical simulation^14^. The formula to calculate *D*_*c*_ is described as *D*_*c*_/*D*_*x*_ = *f*(*n*)/(*f*(*n*) + *N* − 2), where *f*(*n*) = 1.86 + 1.08 *n* + 1.38 *n*^2^ ^14^. (**b**) All solid triangles are particles behaving *zigzag mode*, while hollow ones *displacement mode*; downward ones in 3000 ppm PVP solutions, while upward ones in 8000 ppm PVP solutions; red ones in *N* = 5, while black ones in *N* = 8.

Table 1 presents the rheology information of test fluids. Aqueous Xanthan solutions are strongly shear-thinning fluid (see Fig. 2(a)) without significant normal stress difference observed^36^ and PVP solutions has a constant viscosity at a certain concentration but with elasticity (see Fig. 2(b,c)). This helps us to isolate the effects of shear-thinning and elasticity of non-Newtonian fluid medium. Xanthan Gum solutions were modeled by power-law fluid and each power law index *n* indicates a different concentration of Xanthan Gum solution. PVP solutions are Boger type, with constant viscosity (*η*) during decades of shear rate, and its remarkable elasticity is characterized by relaxation time (*λ*). Figure 3 illustrates the dynamic range of *D*_*c*_ of DLD devices with *N* = 5 and 8 for different fluids. Although displacement angle *φ* for *displacement mode* is *θ*, there remains displacement angle *φ* (0 < *φ* < *θ*) where particles don’t either behave *zigzag mode*. However, the geometry parameters chosen here (*D*_*y*_/*D*_*x*_ = 10/3) guarantees the symmetry of the flow lane distribution and meanwhile avoids “mixed motion”, i.e., particle trajectory with a displacement angle *φ* (0 < *φ* < *θ*)^37^. Moreover, the intermediary angle is short in this paper and have little influence on particle separation in DLD. It is also unrealistic to separate particles with similar size by hydrodynamic forces. We hence note that particles whose displacement angle is over zero have entered *displacement mode*. After superposition of over 10,000 images captured by the camera via Z Project in ImageJ, examples of which can be seen in Fig. 1(c,d), the mode of particles entering a certain mode are determined, either *zigzag* or *displacement*. The modes of particle in different circumstances including particle diameter, *N*, *n*, and *Wi* were plotted.

**Table 1:** Rheology information of test fluids: power law fitting parameter of viscosity versus shear rate of Xanthan Gum solution, and constant viscosities and relaxation times of PVP solutions as a Boger fluid.

Solutions	Concentration	Power law fit (${\boldsymbol{\eta }}={\boldsymbol{m}}{\dot{{\boldsymbol{\gamma }}}}^{{\boldsymbol{n}}-{\bf{1}}}$)		*η*(Pa · s)	*λ*(s)
		*m*	*n*		
Xanthan gum	700 ppm	0.090	0.685		
	1000 ppm	0.149	0.608		
	1500 ppm	0.359	0.526		
PVP	3000 ppm			0.155	2.15 × 10 ^– 3^
PVP	8000 ppm			0.385	2.57 × 10^−3^

**Figure 4:**
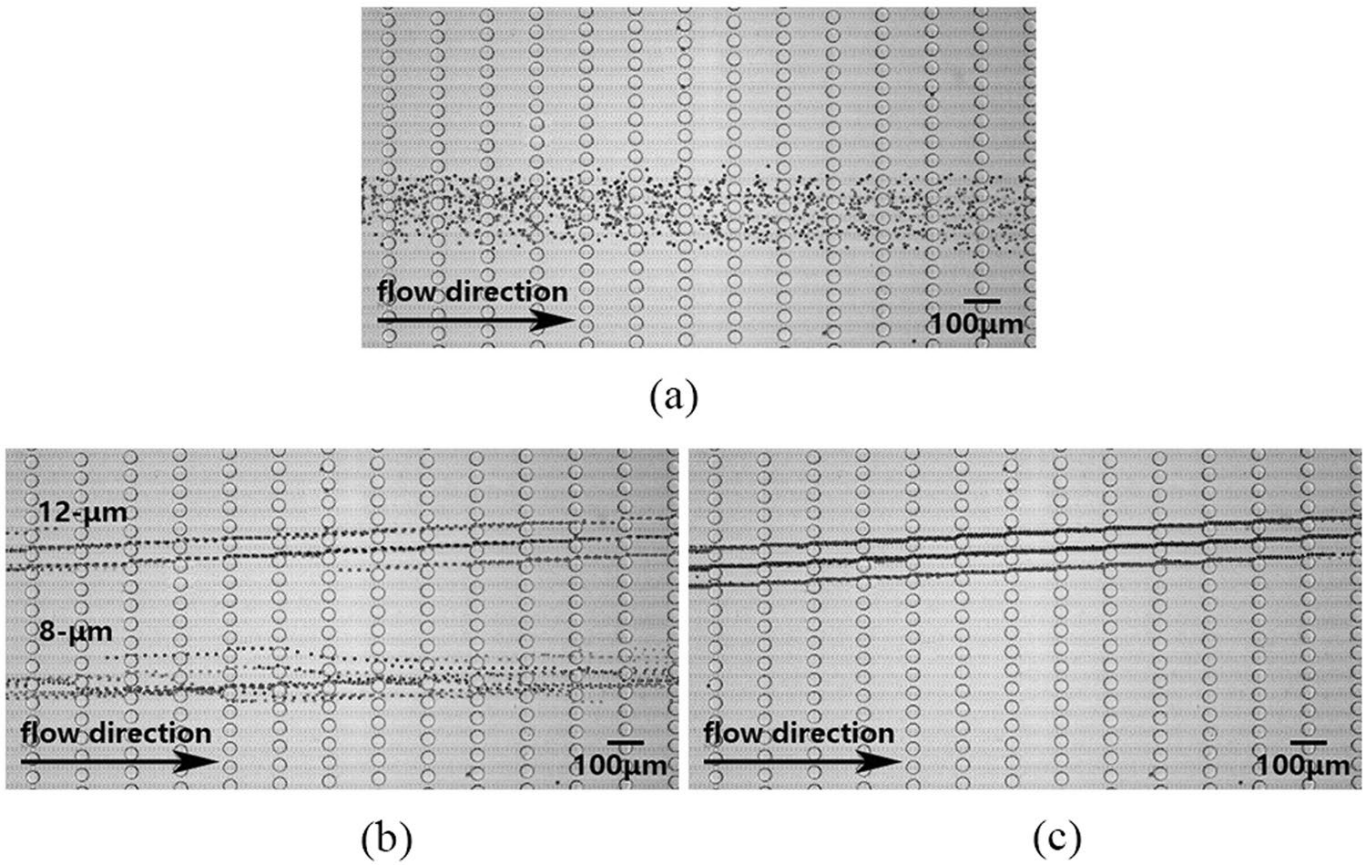
Dynamic control of particle separation in viscoelastic DLD via flow rate at (**a**) *Wi* = 0.1, (**b**) *Wi* = 0.5, and (**c**) *Wi* = 0.9. (**a**) Particles with diameter 8 *μm* and 12 *μm* behave zigzag mode at low *Wi*, i.e., low flow rate; (**b**) 12-*μm* particles enter displacement mode while 8-*μm* particles remain unchanged when the flow rate gradually increases; (**c**) Both 12-*μm* and 8-*μm* particles finally enter displacement mode over a critical flow rate. In this way, *D*_*c*_ can be tuned by flow rate in viscoelastic DLD. The images are superposed over the Supplementary Video S3.

In order to illustrate the dynamic control of particle separation in viscoelastic DLD devices, we present the separation of particles with diameter 8 *μm* and 12 *μm* in Fig. 4 and Supplementary Video S3. At first, both 8-*μm* and 12-*μm* particles behave zigzag mode at *Wi* = 0.1, i.e., low flow rate. The critical size at this situation is around 13 *μm*. With the flow rate gradually increasing, *D*_*c*_ declines due to the increased *Wi*. 12-*μm* particles enter displacement mode once *D*_*c*_ decreases under 12 *μm* while 8-*μm* particles remain unchanged since *D*_*c*_ is still over 8 *μm* at approximately *Wi* = 0.5. At this situation, we realize separation of particles with two diameters by tuning flow rate despite that they cannot be separated in Newtonian DLD devices with the same geometry and flow rate. With the flow rate further increasing, both 8-*μm* and 12-*μm* particles enter diaplacement mode presented as Fig. 4(c).

## Discussion

The separation threshold versus shear-thinning effect is presented in Fig. 3(a). It is observed that *D*_*c*_ declines with *n* decreasing, which implies that we can separate particles with different critical sizes in one exact DLD device, by only changing the shear-thinning fluid medium with different concentrations. The experimental results are basically in consistence with the dashed lines that were provided by D’Avino^14^ with numerical results.

The basic principle of particle separation in DLD can be understood by the streamline orientation of DLD arrays. Fluid emerging from a gap between two obstacles will encounter another one in the next row and will bifurcate as it moves around the obstacle. As this process repeats, periodical bifurcation of the fluid results in the *N* regions returning to their original relative position with the single gap. Each region entrains the same amount of fluid with the others and carries the same group of molecules following the same path throughout the array. Therefore, David *et al*.^9^ derived a formula *D*_*c*_ = 2*αD*_*x*_/*N* to calculate theoretical *D*_*c*_. In this formula, parameter *α* denotes the non-uniformity of the flow through the gap. If the fluid flow velocity profile between the two posts is plug-like, *α* = 1; if the flow velocity profile is parabolic, $\alpha =\sqrt{N\mathrm{/3}}$ demonstrated by Beech^38^, considering practical reality. Fluid flow with different shear-thinning effect corresponds to a different *α*, and consequently *D*_*c*_. The modification of *D*_*c*_ in shear-thinning fluids is due to that the shear-thinning effect flats the parabolic velocity profile between the posts nearby and thus the width of the outermost flow lane, become larger than that in Newtonian fluids^14^. The thinner the shear of the fluid is, the smaller *α* is. In our experiments, the maximum value of the relative difference of *D*_*c*_ with power-law and Newtonian fluids is found to be around 40% (i.e., when *N* = 8, *D*_*c*_ ≈ 12.3 *μm* in Newtonian fluids, whereas *D*_*c*_ ≈ 7.1 *μm* in a 2000 ppm Xanthan solution). Note that changing the fluid medium still seems to be complicated to alter *D*_*c*_ in a single DLD device.

We then employ PVP solutions as our testing fluids. As PVP solutions behave like a Boger fluid^39^, they allow us to investigate the elastic effects of viscoelastic fluids solely. Figure 3(b) illustrates the motion modes of particles with different sizes under different Weissenberg number (*Wi* = *λu*/*D*_*x*_, where *u* is average velocity when the fluid flows through the gap between neighbor posts, and in the limiting case, *Wi* in the Newtonian case is regarded as zero). Considering that each inlet has the same area of the cross section and flow rate, *u* is obtained by dividing the flow rate by the area of the cross section between two posts to calculate *Wi*. The *Wi* number indicates the strength of elastic effect on the flow, and it is in a positive linear relationship with the flow rate. It is clear that, in the two DLD arrays with *N* = 5 and 8, *D*_*c*_ in PVP solutions becomes smaller than that in Newtonian fluids. And *D*_*c*_ decreases along with the increase of *Wi*. The fit between *D*_*c*_ and *Wi* seems to be linear for the same *N* (the dashed line nipped by *displacement* and *zigzag mode* in Fig. 3(b)). The above finding implies that although both the DLD array and the fluid medium are fixed, we can still tune *D*_*c*_ of a DLD device by changing *Wi*, i.e., the flow rate at the inlet, to achieve dynamic control of particle separation.

In order to explain the abnormal change of *D*_*c*_ with the Boger fluid, we take into account the first normal stress difference of the viscoelastic fluid flow. In a viscoelastic Poiseuille flow, suspended particles will laterally migrate towards specific equilibrium positions due to the non-uniform *N*_1_ and the second normal stress difference *N*_2_. *N*_2_ is usually neglected because of its relatively small magnitude (~*O*(10^−2^)) comparing with *N*_1_^40^. The elastic lift force **F**_**e**_ in inertial microfluidics can be expressed as ${{\bf{F}}}_{{\bf{e}}}\propto {a}^{3}\nabla {N}_{1}$^33^. The elastic force arising from the non-uniform *N*_1_ plays a more significant role than other inertial lift forces when elasticity is dominant. Especially in a pure elastic flow, particle will migrate towards the centerline in a circular tube and another four corners in a channel with a square cross section, where *N*_1_ is lower than other regions^33^. Inspired by the elastic force arising from *N*_1_ in inertial microfluidics, we attribute the decrease of *D*_*c*_ in the Boger fluid to the appearance of non-uniform *N*_1_. The application of an elastic lift force pushes the particle out from the post into the neighboring lamina, displacing the particle despite that its size is smaller than the critical size in the Newtonian case. Therefore, in a DLD device, the transition from the zigzag motion to the *displacement mode* is advanced by extra elastic force with the particle size getting increased. Numerical simulations of single-phase viscoelastic elastic fluid flow passing through periodic obstacles are simulated to demonstrate the distribution of *N*_1_. Since particles suffer the periodic forces in every unit, we performed two-dimensional numerical simulations on fluid flow in a unit of DLD array (Fig. 1(b)) via *OpenFOAM*^41^.

**Figure 5:**
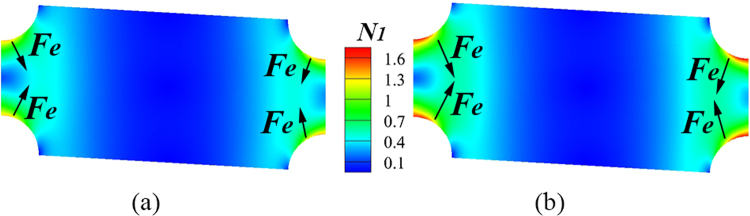
Contour of *N*_1_ over a unit of a DLD array at (**a**) *Wi* = 0.2 and (**b**) *Wi* = 10. The arrows represent the direction of elastic force **F**_**e**_.

Figure 5 plots the contour of *N*_1_ in one unit at *Wi* = 0.2 and 10. The elastic force pushes particles towards lower *N*_1_ region, whose direction are presented along the arrow in Fig. 5. Moreover, the gradient of *N*_1_, $\nabla {N}_{1}$, at high *Wi* is greater than that at small *Wi*, and consequently a large elastic force is exerted on the particle. That’s why *D*_*c*_ becomes smaller when *Wi* increases. It can also be understood by shell model induced by irreversible non-hydrodynamic interactions^42^. The elastic force arising from the non-uniform *N*_1_ enlarge the hard-wall potential of the model. The current work does not enter the further higher *Wi* region. For even high *Wi*, much lower *D*_*c*_ may be allowed. However, the strength of the microchannel cannot meet the demand for higher pressure drop as the viscosity in PVP solutions is 2 order higher than that in Newtonian fluids. It will be valuable to search for a typical elastic fluid with a lower viscosity and strong elasticity in the future.

In summary, we investigated how the critical separation size *D*_*c*_ of the deterministic lateral displacement (DLD) device is influenced by non-Newtonian fluids, i.e., the shear-thinning effect and the elastic effect. For the first time, dynamic control of *D*_*c*_ can be easily realized through only tuning the flow rate in microchannels. Our experimental results show that both the shear-thinning and elastic effects can be used to tune *D*_*c*_ of a DLD array. It is found that *D*_*c*_ decreases when power law index n decreases or *Wi* increases. The maximum reduction of modified *D*_*c*_ in the experiments with non-Newtonian fluids over *D*_*c*_ in Newtonian fluids is up to 40% approximately. The variation of *D*_*c*_ under shear-thinning effect is attributed to a flatter velocity profile between two neighboring posts. We believe the extra elastic force arising from the non-uniform first normal stress difference *N*_1_ is responsible for the reduction of *D*_*c*_ in elastic fluid medium. Moreover, larger *Wi* provides larger $\nabla {N}_{1}$ between the posts so that *D*_*c*_ declines more rapidly than that at low *Wi*. In this manner, a new dynamic approach to tuning *D*_*c*_ in a DLD array is proposed: the flow rate of a DLD array can be utilized to tune *D*_*c*_ in viscoelastic fluid medium. In a DLD device, although *D*_*c*_ in Newtonian fluids is fixed, we can change the fluid medium with different shear-thinning strength to alter *D*_*c*_. Adopting viscoelastic fluid offers a new opportunity of dynamically tuning *D*_*c*_ by changing the flow rate, which can greatly simplify the existing methods of particle separation control in DLD devices without introducing any auxiliary equipment.

## Methods

### Microchannel Fabrication and Design

**Figure 6:**
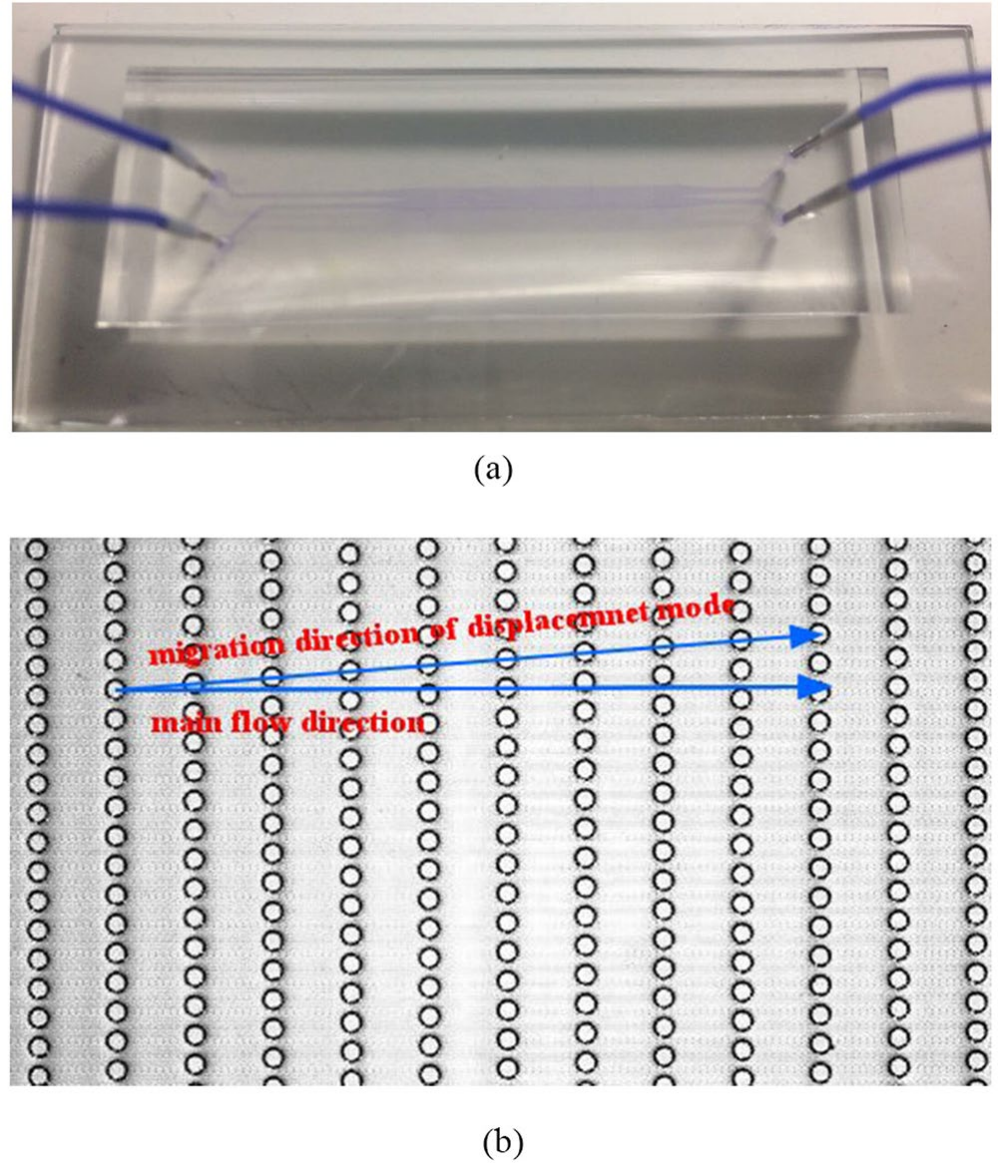
(**a**) A snapshot of the microfluidic channel with poly(dimethylsiloxane) (PDMS)-glass compounded layer field with purple dye. (**b**) A grey-scaled image of micro-posts in the microchannel. The direction of main flow and displacement mode are presented.

The microfluidic channel was fabricated by the soft lithography techniques using poly(dimethylsiloxane) (PDMS)-glass compounded layer, as shown in Fig. 6. Liquid PDMS was prepared by mixing pre-polymer (Sylgard 184, Dow Corning, USA) with the curing agent by the weight ratio of 10:1. Once both liquid components are thoroughly cross-linked and degassed, PDMS was cast over the SU8 (MicroChem, Newton, MA, USA) master mold on a silicon substrate and then was baked in an oven at 80 °C for 1 hour. After the PDMS was peeled off from the channel mold, several holes were punched through the PDMS slab according to the reserved circles in the microchannel serving as reservoirs of inlets and outlets. The PDMS slab was then treated with oxygen plasma (Harrick, USA) and bonded to a glass substrate. The plastic tubes were inserted through these ports and the tubes were sealed at the junction with the PDMS slab using the glue. Finally, the PDMS-glass assemble device was placed into an oven at at 80 °C for 30 minutes to enhance the bonding. The geometry parameters chosen here: *N* = 5 or 8, *D*_*x*_ = 30 *μ*m, *D*_*p*_ = 50 *μ*m and *D*_*y*_ = 10/3 × *D*_*x*_, which guarantees the symmetry of the flow lane distribution and meanwhile avoids “mixed motion”^37^.

### Working Fluids Preparation

The shear-thinning Xanthan solution was prepared by adding Gum Xanthan (*Mw* = 4 × 10^6^ g/mol, Sigma-Aldrich, USA) powder to 22 wt% glycerin (Sigma-Aldrich, USA) aqueous solution (deionized water) to match the density of the polystyrene (PS) particles (1.05 × 10^3^ kg/m^3^). The viscoelastic Boger fluid^39^ was prepared by adding polyvinylpyrrolidone (PVP) (*Mw* = 3.6 × 10^5^ g/mol, Sigma-Aldrich, USA) to 22 wt% glycerin (Sigma-Aldrich, USA) aqueous solution. The sample liquid was made by adding polystyrene (PS, Applied Microspheres, the Netherlands) particles into buffer liquid with 0.05%wt Tween 20 (Sigma-Aldrich, USA), which was used to prevent particles’ aggregation. All solutions were well stirred for 24 hours and kept for another 24 hours. The volume fraction of particles in the suspension is 0.003. Table 2 presents the particle diameters and their error bars. The viscosity versus shear rate and dynamic oscillation of elastic and viscous modulus was measured by a rotational rheometry (Kinexus, Malvern Instruments Ltd.) equipped with cone-plate geometry (diameter *d* = 60 mm, cone angle *α* = 1°) at *T* = 298 K. The instrument operates in a strain-controlled mode and all frequency sweeps were done at strain amplitudes *γ*_0_ < 100% to assure the linear viscoelastic response regime. The characteristic shear relaxation time *λ* is calculated from the low frequency part of the data according to $\lambda =\mathop{\mathrm{lim}}\limits_{\omega \to 0}\frac{G^{\prime} }{G^{\prime\prime} \omega }$, where the limiting scaling relations satisfy $G^{\prime} \sim {\omega }^{2}$ and $G^{\prime\prime} \sim \omega$.

**Table 2:** The diameters of particles with error bars adopted in this experiments.

Particle diameter *a* (*μ*m)	7.1	8.2	8.9	10.1	11.2	12.3	13.4
Error bar (*μ*)	±0.28	±0.23	±0.22	±0.19	±0.19	±0.20	±0.21
Particle diameter *a* (*μ*m)	14.2	14.695	15.3	16.1	16.8	17.8	18.9
Error bar (*μ*)	±0.19	±0.15	±0.14	±0.15	±0.14	±0.15	±0.16

### Experimental Procedures and Image Analysis

Experimental liquids were injected into the microchannels in a 1-mL syringe (Hamilton, Switzerland) with a syringe pump (Harvard, USA). The chip was mounted on the stage of an inverted microscope (IX71, Olympus, Japan), the motion was captured by a high-speed camera (Phantom v.73, Vision Research Inc., USA) with the rate of 100 images per second and the images were analyzed utilizing the ImageJ software (Fiji, ImageJ 1.51 n).

### Numerical Method

The simulations were performed using *OpenFOAM* (open source CFD software, *OpenFOAM*-extend 3.2) which is based on Finite Volume Method (FVM)^43^. The governing equations are made dimensionless by taking the gap *D*_*x*_ as characteristic length, the maximum velocity um as characteristic velocity, the viscosity *η*_0_ as characteristic viscosity. Denoting with starred symbols the dimensionless quantities, the fluid flow was simulated in the device by solving the incompressible Navier-Stokes and continuity equations:

1 $$\frac{\partial {{\bf{u}}}^{\ast }}{\partial {t}^{\ast }}+{{\bf{u}}}^{\ast }\cdot {\nabla }^{\ast }{{\bf{u}}}^{\ast }=-\frac{{\nabla }^{\ast }{p}^{\ast }}{{\rho }^{\ast }}+{\nabla }^{\ast }\cdot {\tau }^{\ast },{\nabla }^{\ast }\cdot {{\bf{u}}}^{\ast }=0,$$

where τ^*^ is the dimensionless total stress, which can be written split into polymeric (viscoelastic) part separately and the solvent part. Therefore, the momentum balance equations for Oldroyd-B model is

2 $$\frac{\partial {{\bf{u}}}^{\ast }}{\partial {t}^{\ast }}+{{\bf{u}}}^{\ast }\cdot {\nabla }^{\ast }{{\bf{u}}}^{\ast }=-\frac{{\nabla }^{\ast }{p}^{\ast }}{{\rho }^{\ast }}+\frac{\beta }{Re}{\nabla }^{\ast 2}\cdot {{\bf{u}}}^{\ast }+\frac{1-\beta }{ReWi}({\nabla }^{\ast }\cdot ({\bf{C}}-{\bf{I}})),$$

**Figure 7:**
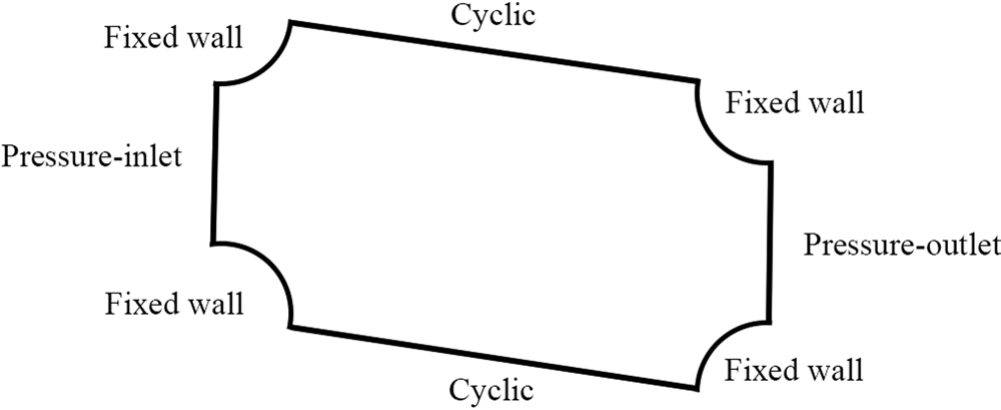
Boundary conditions of a 2-D unit of a DLD array in numerical simulations.

**C** is the conformation tensor of polymer molecules or surfactant micelles and *Re* and *Wi* are dimensionless numbers defined as *Re* = *ρU*_*m*_*D*_*x*_/*η*_0_ and *Wi* = *λU*_*m*_/*D*_*x*_, which represent inertial forces versus viscous forces and elastic forces versus viscous forces, respectively. We employ log-conformation algorithm to solve High Weissenberg Non-linear problem (HWNP), the details and the validation of which can be referred to our previous paper^41^. Numerical model with its boundary conditions is presented in Fig. 7.

## Electronic supplementary material

Supplementary Video 1

Supplementary Video 2

Supplementary Video 3
