# Supplementary figures and images for: Dynamic control of particle separation in deterministic lateral displacement separator with viscoelastic fluids
Source: Sci Rep. 2018 Feb 26;8:3618. doi: 10.1038/s41598-018-21827-7 (PMC5827740; doi:10.1038/s41598-018-21827-7)

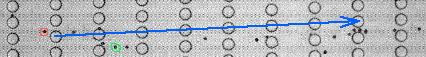

Supplement: Supplementary file 1 — Supplementary Video 1 [file 41598_2018_21827_MOESM1_ESM.gif]

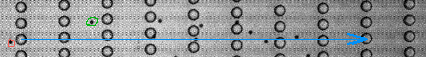

Supplement: Supplementary file 2 — Supplementary Video 2 [file 41598_2018_21827_MOESM2_ESM.gif]
